# Bioactive Constituents and Antihypertensive Mechanisms of Zhengan Xifeng Decoction: Insights from Plasma UPLC–MS, Network Pharmacology and Molecular Dynamics Simulations

**DOI:** 10.3390/ph18101493

**Published:** 2025-10-04

**Authors:** Yu Wang, Yiyi Li, Zhuoying Lin, Niping Li, Qiuju Zhang, Shuangfang Liu, Meilong Si, Hua Jin

**Affiliations:** 1 School of Basic Medicine, Gansu University of Chinese Medicine, Lanzhou 730000, China; wangyugansu@126.com (Y.W.); zqj@gszy.edu.cn (Q.Z.); 2 Key Laboratory of Dunhuang Medicine, Ministry of Education, Lanzhou 730000, China; doublelsf@163.com (S.L.); gslzsml@163.com (M.S.); 3 Key Laboratory of Traditional Chinese Herbs and Prescription Innovation and Transformation of Gansu Province, Lanzhou 730000, China; 4 College of Pharmacy, Jinan University, Guangzhou 510632, China; holiyiyi123@stu2022.jnu.edu.cn (Y.L.); zoelim@stu2023.jnu.edu.cn (Z.L.); linipinganan30@jnu.edu.cn (N.L.); 5 Clinical of Traditional Chinese Medicine, Gansu University of Chinese Medicine, Lanzhou 730000, China

**Keywords:** Zhengan Xifeng decoction, hypertension, network pharmacology, molecular docking, molecular dynamics simulation, traditional Chinese medicine

## Abstract

**Background/Objectives**: Hypertension is a global health challenge. Zhengan Xifeng Decoction (ZXD), a classical traditional Chinese medicine, has shown clinical efficacy against hypertension. This study aimed to identify the bioactive constituents of ZXD and elucidate its antihypertensive mechanisms by integrating plasma UPLC–MS (ultra-performance liquid chromatography–mass spectrometry) analysis, network pharmacology, and molecular dynamics (MD) simulations. **Methods**: ZXD constituents and plasma-absorbed compounds were characterized by UPLC–MS. Putative targets (TCMSP, SwissTargetPrediction) were cross-referenced with hypertension targets (GeneCards, OMIM) and analyzed in a STRING protein–protein interaction network (Cytoscape) to define hub targets, followed by GO/KEGG enrichment. Selected protein–ligand complexes underwent docking, Prime MM-GBSA calculation, and MD validation. **Results**: A total of 72 absorbed components were identified, including 14 prototype compounds and 58 metabolites. Network pharmacology identified ten key bioactive compounds (e.g., liquiritigenin, isoliquiritigenin, and caffeic acid), 149 hypertension-related targets, and ten core targets such as SRC, PIK3CA, PIK3CB, EGFR, and IGF1R. Functional enrichment implicated cardiovascular, metabolic, and stress-response pathways in the antihypertensive effects of ZXD. Molecular docking demonstrated strong interactions between key compounds, including liquiritigenin, caffeic acid, and isoliquiritigenin, and core targets, supported by the MM-GBSA binding free energy estimation. Subsequent MD simulations confirmed the docking poses and validated the stability of the protein–ligand complexes over time. **Conclusions**: These findings provide mechanistic insights into the multi-component, multi-target, and multi-pathway therapeutic effects of ZXD, offering a scientific basis for its clinical use and potential guidance for future drug development in hypertension management.

## 1. Introduction

Hypertension is a major global health challenge, affecting over 1 billion adults and markedly increasing the risk of cardiovascular disease, which remains the leading cause of premature mortality worldwide [1]. A large-scale analysis of 1201 population-based studies, involving over 104 million participants, revealed that the number of individuals aged 30–79 with hypertension doubled from 1990 to 2019, increasing from 331 million women and 317 million men to 626 million women and 652 million men [2]. In China, the prevalence of hypertension has escalated sharply over the past three decades and now impacts nearly a quarter of the adult population [3]. This growing public health burden underscores the urgent need for effective and sustainable therapeutic strategies that address the underlying mechanisms of hypertension rather than solely managing symptoms.

Traditional Chinese medicine (TCM) offers unique therapeutic advantages in hypertension management owing to its multi-component, multi-target, and multi-pathway characteristics. TCM formulations have demonstrated efficacy in lowering blood pressure, protecting target organs, and reducing the complications associated with hypertension through synergistic interactions among their constituent herbs [4,5]. Zhengan Xifeng Decoction (ZXD), a classical TCM formulation documented by Zhang Xichun in Yixue Zhongzhong Canxilu, consists of twelve medicinal herbs (see graphical abstract for a visual summary; full list in Section 4.1) traditionally prescribed to calm liver yang, suppress internal wind, and nourish yin [6,7]. Clinically, ZXD has been widely applied in the treatment of essential hypertension [8,9]. Substantial evidence has demonstrated that ZXD significantly reduces both systolic and diastolic blood pressure, improves clinical efficacy, enhances vascular endothelial function, alleviates hypertension-related symptoms, and maintains a favorable safety profile with minimal adverse drug reactions [10,11,12]. In addition, animal studies and our previous experimental investigations have consistently confirmed ZXD’s significant antihypertensive effects [13,14,15,16,17,18]. However, the specific bioactive components and their underlying mechanisms of action remain largely unexplored.

Network pharmacology, a comprehensive approach integrating systems biology, computational modeling, and pharmacology, provides a robust framework for unraveling the multifaceted interactions within complex TCM formulations and disease pathways [19,20]. This approach, when combined with molecular docking, facilitates the identification of bioactive compounds and target proteins, thereby elucidating the mechanisms underlying multi-component, multi-target therapies [21,22]. Molecular docking further allows prediction of binding affinities between compounds and their targets, which is essential for clarifying the contributions of individual constituents within complex TCM prescriptions [23]. Moreover, MD simulations provide dynamic insights into protein–ligand interactions by evaluating conformational stability, binding kinetics, and free energy landscapes [24].

In this study, we integrated plasma UPLC–MS analysis with network pharmacology, molecular docking, and MD simulations to systematically investigate the bioactive constituents and antihypertensive mechanisms of ZXD. By identifying key compounds, targets, and signaling pathways, our work aims to provide mechanistic insights into the multitarget therapeutic actions of ZXD and a scientific basis for its clinical application in hypertension management.

## 2. Results

### 2.1. Characterization of ZXD Chemical Constituents and Absorbed Compounds

**Figure 1:**
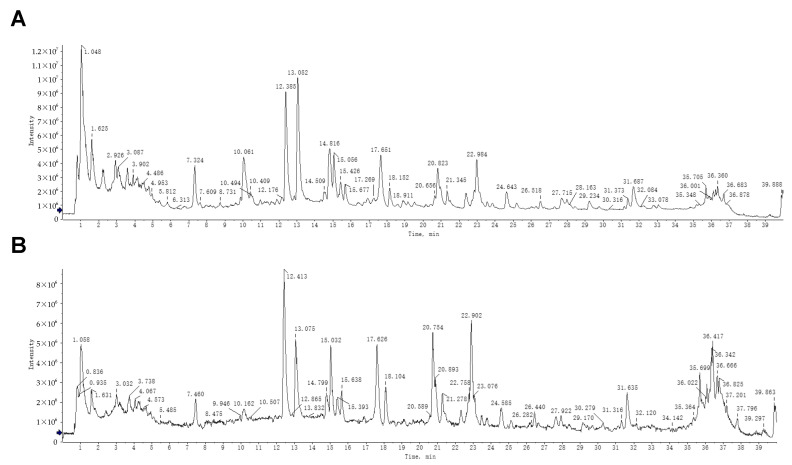
UPLC–MS analysis of ZXD. (**A**) Total ion chromatogram of ZXD in negative ion mode. (**B**) Total ion chromatogram of ZXD in positive ion mode.

Extensive pharmacological investigations, including our prior studies, have established that ZXD demonstrates significant antihypertensive activity [7,13]. To elucidate the mechanistic basis of its therapeutic effects, systematic identification of bioactive constituents is essential. In this study, UPLC–MS was employed for an in-depth chemical characterization of ZXD. Total ion chromatograms were acquired in both positive and negative ion modes (Figure 1), enabling the detection of compounds with differing ionization efficiencies. This dual-mode acquisition broadened the coverage and provided detailed insight into both major and minor constituents.

To ensure analytical rigor, the observed *m*/*z* values and fragmentation patterns were compared with those of compounds previously reported in the literature. As a result, 113 chemical constituents were identified, of which 13 compounds were newly identified in ZXD in this study [17]. Detailed information about these compounds, including retention time (RT), ion mode, *m*/*z* values, mass errors, molecular formulas, and fragment ions, is presented in Table 1.

**Figure 2:**
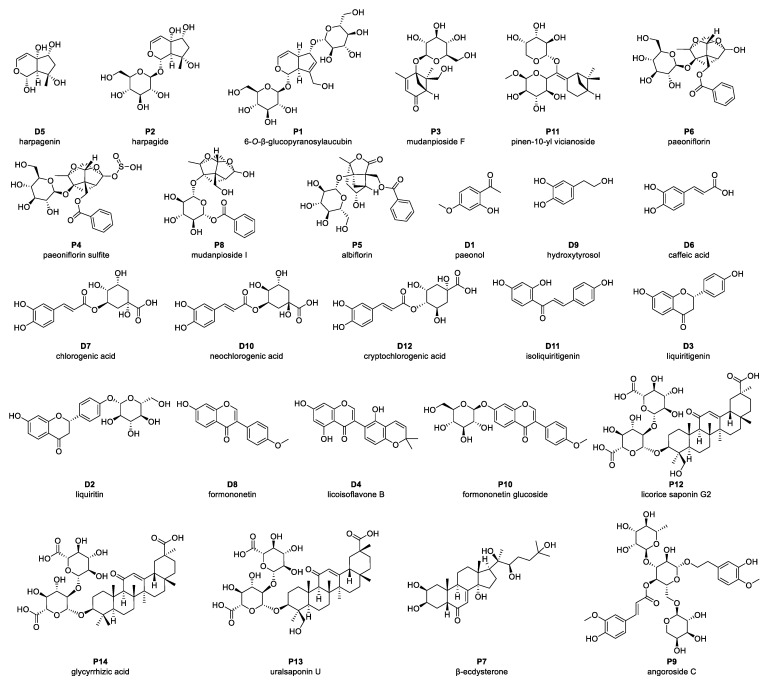
Prototype compounds identified in plasma following administration of ZXD using UPLC–MS. Compounds labeled “P” denote prototype constituents detected in both the ZXD and plasma; compounds labeled “D” denote metabolites derived from these prototype constituents during in vivo ZXD metabolism.

In addition to identifying constituent compounds, it is crucial to assess which of these compounds are absorbed into the systemic circulation, as only bioavailable compounds can exert physiological effects in vivo [25]. To achieve this, plasma samples were collected following oral administration of ZXD, and the absorbed compounds were identified using UPLC–MS (Figure S1). A total of 72 compounds were detected in plasma, comprising 14 prototype compounds (Figure 2, Table S1) and 58 metabolites (Table S2). The corresponding prototype compounds for these metabolites are illustrated in Figure 2. These detected metabolites were predominantly generated through metabolic processes such as hydroxylation, sulfation, glucuronidation, hydrolysis, methylation, demethylation, and debenzoylation, reflecting the complex biotransformation pathways of ZXD in vivo.

### 2.2. ZXD Absorbed Compounds–Disease Target Network

To elucidate the pharmacological mechanisms by which ZXD acts against hypertension, a target prediction analysis was conducted on the absorbed components detected in plasma. The SMILES format structures and descriptor data of 26 absorbed components of ZXD, comprising 14 prototype compounds and 12 additional prototype compounds corresponding to 58 metabolites, were retrieved from the PubChem database [26]. Target prediction was performed using the Swiss Target Prediction [27] and TCMSP [28] databases, identifying 422 potential protein targets associated with these constituents.

**Figure 3:**
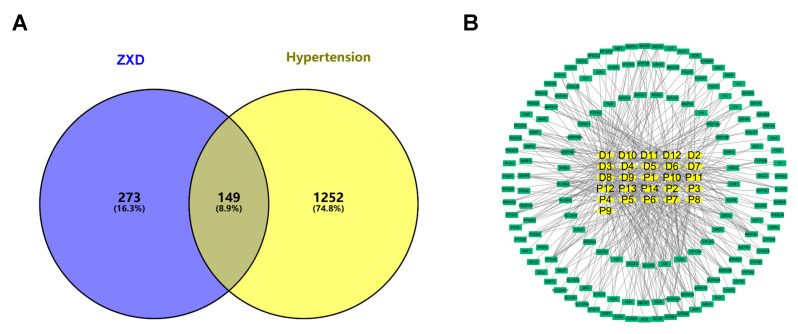
Network pharmacology of ZXD absorbed constituents and hypertension targets. (**A**) Venn diagram showing the overlap between the absorbed components and hypertension-related targets; (**B**) Compound–target network: yellow squares, absorbed constituents; green squares, disease targets.

To refine disease relevance, a comprehensive search was conducted in the GeneCards database [29] using the keyword “hypertension” which yielded 12,366 potential targets. After filtering threshold (≥3) was applied, 1401 unique targets were retained. An additional 34 hypertension-related genes were retrieved from OMIM [30]. Intersecting the 422 constituent-associated targets with the 1401 disease targets produced 149 common targets (Figure 3A). These were visualized in the compound–target network using Cytoscape 3.10.2 [31]. Key topological parameters, including degree, average shortest path length (ASPL), and betweenness centrality (BC), were used to evaluate node importance (Figure 3B). Network analysis prioritized ten key bioactive constituents, such as isoliquiritigenin, liquiritigenin, formononetin, and caffeic acid, based on centrality and their interactions with hypertension-related targets (Table 2), indicating that these compounds may act as active constituents of ZXD in the treatment of hypertension.

### 2.3. Protein–Protein Interaction (PPI) Network Construction and Topological Analysis

**Figure 4:**
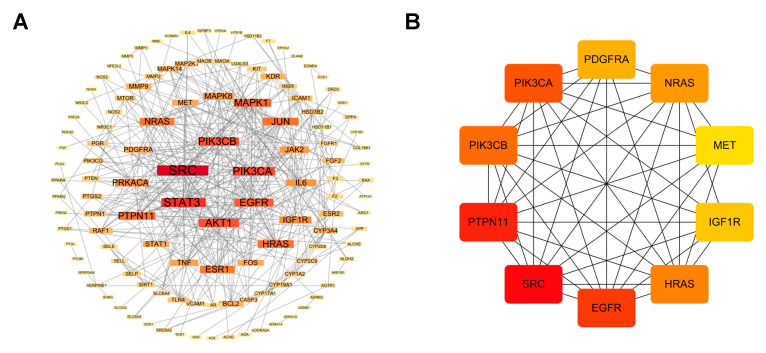
Protein–protein interaction network construction and core targets. (**A**) High-confidence protein–protein interaction network for the 149 common targets; (**B**) Ten core targets prioritized by CytoHubba (MCC). Red denotes higher hubness (top MCC–ranked nodes); yellow indicates lower hubness.

The 149 common targets identified between ZXD’s absorbed compounds and hypertension-related targets were further analyzed to elucidate their interactions at the protein level. Protein–protein interaction was retrieved from the STRING database [32] with a confidence threshold of >0.9 to ensure high specificity. This resulted in a PPI network consisting of 137 nodes and 370 edges. This yielded a PPI network comprising 137 nodes and 370 edges. The high-confidence network was subsequently visualized using Cytoscape (Figure 4A).

To identify the most influential targets, topological properties were assessed using the CytoHubba plugin (MCC algorithm). A red-to-yellow color gradient was applied in Cytoscape to indicate hub nodes, reflecting their relative importance. Using CytoHubba (MCC), we prioritized the top 10 hub targets—SRC, PTPN11, EGFR, PIK3CA, PIK3CB, HRAS, NRAS, PDGFRA, IGF1R, and MET—as core targets (as defined in Methods) (Figure 4B). Among these, the PI3K/AKT pathway, represented by PIK3CA and PIK3CB, emerged as a critical regulator of endothelial function, vascular smooth muscle cell proliferation, and inflammation, all processes that are essential for blood pressure regulation [33,34].

### 2.4. GO and KEGG Enrichment of Common Targets

**Figure 5:**
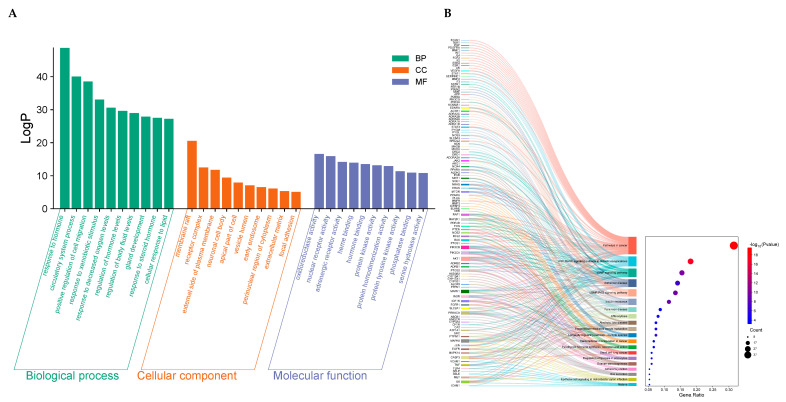
GO and KEGG enrichment analysis. (**A**) GO enrichment analysis of the 149 common targets; (**B**) Top 20 KEGG pathway enrichments for the 149 common targets.

To gain insight into the biological roles of the 149 intersecting targets, we performed Gene Ontology (GO) enrichment using Metascape, covering biological processes (BP), cellular components (CC), and molecular functions (MF) categories. As shown in Figure 5A the BP associated with the ZXD targets included response to circulatory system processes, positive regulation of cell migration, response to xenobiotic stimuli, and response to decreased oxygen levels. These BP are critical for cardiovascular health, vascular regulation, and cellular adaptation to stress.

The CC involved encompass membrane rafts, receptor complexes, the external side of the plasma membrane, neuronal cell bodies, apical parts of cells, vesicle lumens, early endosomes, the perinuclear region of the cytoplasm, extracellular matrix, and focal adhesions. For MF, the enriched terms primarily include oxidoreductase activity, nuclear receptor activity, adrenergic receptor activity, heme binding, and hormone binding.

To further clarify the mechanisms by which ZXD may exert antihypertensive effects, KEGG pathway enrichment analysis was performed. The top 20 enriched pathways, ranked by *p*-value, are shown in Figure 5B. These pathways were mainly associated with cardiovascular regulation, metabolic homeostasis, and stress responses. Representative terms included the AGE–RAGE signaling pathway in diabetic complications, the cAMP signaling pathway, the cGMP–PKG signaling pathway, insulin resistance, and regulation of lipolysis in adipocytes. Notably, several core targets of ZXD, including PIK3CA, PIK3CB, and AKT1, were significantly enriched and are known key components of the PI3K/AKT pathway, suggesting that this axis may also contribute to the antihypertensive effects. Collectively, these results indicate that ZXD may lower blood pressure by modulating multiple interconnected pathways governing vascular function, energy metabolism, and inflammatory regulation.

### 2.5. Molecular Docking Results for Key Compounds and Core Targets

Molecular docking was performed to evaluate interactions between the key absorbed components of ZXD and the ten core target proteins from the PPI network. Complexes with binding energies below −6.0 kcal·mol^−1^ were considered to exhibit favorable binding (i.e., stronger predicted affinity and more stable poses) [35], and results meeting this cut-off are summarized in Table 3. Key bioactive compounds, including liquiritigenin, caffeic acid, and isoliquiritigenin, exhibited notable binding affinities with core targets, such as PIK3CA (−8.725 kcal·mol^−1^), IGF1R (−8.689 kcal·mol^−1^), PIK3CB (−7.113 kcal·mol^−1^), and EGFR (−7.055 kcal·mol^−1^).

**Figure 6:**
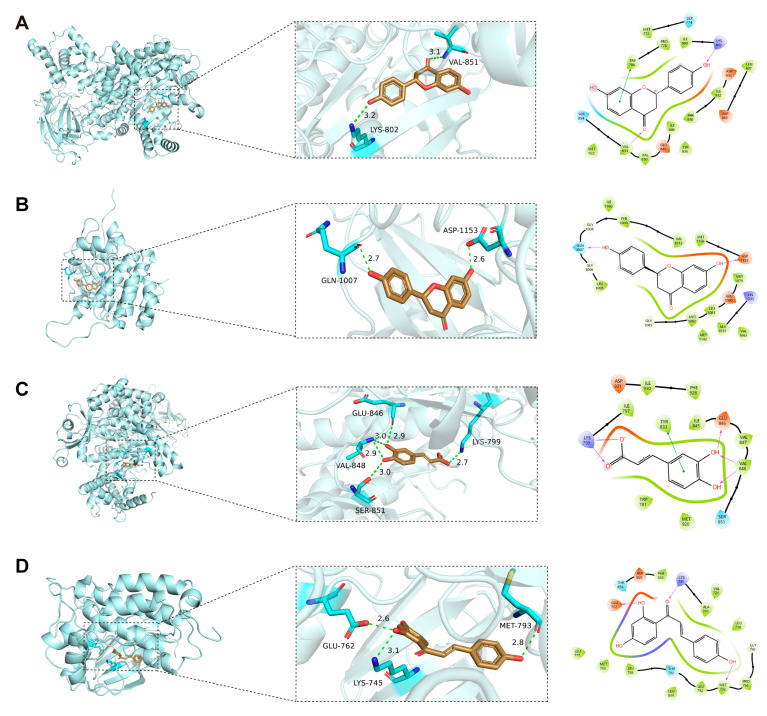
Representative docking poses for four protein–ligand complexes. (**A**) PIK3CA–liquiritigenin. (**B**) IGF1R–liquiritigenin. (**C**) PIK3CB–caffeic acid. (**D**) EGFR–isoliquiritigenin. Three-dimensional overviews, close-up views, and interaction diagrams are shown; visualizations were generated with PyMOL 3.1.6, and interaction diagrams with the Ligand Interaction module in Maestro. For 3D panels (left), protein cartoon: light cyan; ligand carbons: tan; oxygen: red; nitrogen: blue; interacting residues: cyan sticks; hydrogen bonds: green dashed lines with distances (Å). For 2D panels (right), orange: negatively charged residues; purple: positively charged residues; light green: hydrophobic residues; light blue (cyan): polar residues; light gray circles: solvent exposure; solid green lines: π–π stacking interactions; purple dashed arrows: hydrogen bonds with their occupancy percentages.

Visualization of the docking simulations using PyMOL 3.1.6 software revealed the detailed molecular interactions and binding configurations. Four representative complexes (the two best-scoring plus two spanning distinct targets/chemotypes) are shown in Figure 6. Among these, liquiritigenin exhibited the strongest affinity (−8.725 kcal·mol^−1^), occupying the active pocket of PIK3CA. It formed two hydrogen bonds with Lys802 and Val851, along with multiple hydrophobic contacts that markedly stabilized the complex. In addition, a π–π stacking interactions were observed between the aromatic ring of liquiritigenin and Trp780 (Figure 6A). Liquiritigenin also bound strongly to IGF1R (−8.689 kcal·mol^−1^), forming hydrogen bonds with Gln1007 and Asp1153, supplemented by multiple hydrophobic contacts that reinforced complex stability (Figure 6B). Caffeic acid bound to the active site of PIK3CB with a docking score of −7.113 kcal·mol^−1^, forming hydrogen bonds with Lys799, Glu846, and Val848, while Tyr833 established a π–π stacking with the aromatic ring of caffeic acid (Figure 6C). Similarly, isoliquiritigenin showed favorable affinity toward EGFR (−7.055 kcal·mol^−1^), where a combination of hydrogen bonding and hydrophobic interactions contributed to the stabilization of the protein–ligand complex (Figure 6D).

### 2.6. MM-GBSA Binding Free Energies for Representative Protein–Ligand Complexes

**Figure 7:**
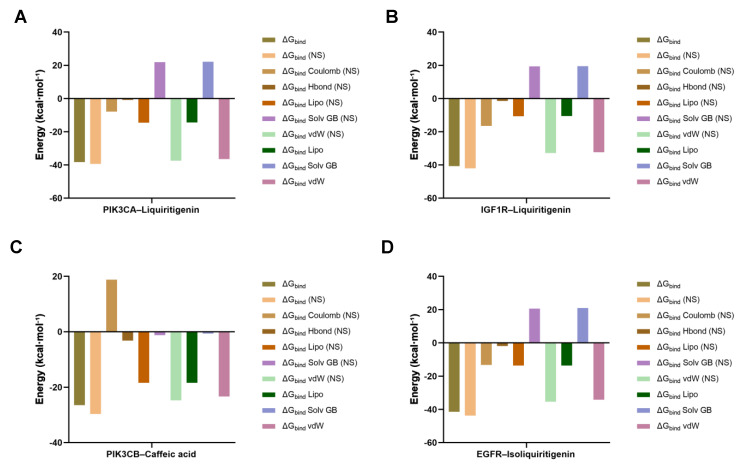
MM-GBSA binding free energy decomposition profiles of four protein–ligand complexes. (**A**) PIK3CA–liquiritigenin; (**B**) IGF1R–liquiritigenin; (**C**) PIK3CB–caffeic acid; (**D**) EGFR–isoliquiritigenin.

To corroborate the docking poses, binding free energies (Δ*G*_bind_) were evaluated using Prime MM-GBSA. All four complexes exhibited favorable, negative Δ*G*_bind_ values (PIK3CA–liquiritigenin: −38.26 kcal·mol^−1^; IGF1R–liquiritigenin: −40.69 kcal·mol^−1^; PIK3CB–caffeic acid: −26.44 kcal·mol^−1^; and EGFR–isoliquiritigenin: −41.37 kcal·mol^−1^), supporting stable ligand engagement (Figure 7). Energy decomposition indicated that van der Waals and lipophilic terms predominated in stabilizing the complexes, while electrostatic interactions and hydrogen bonding provided additional favorable contributions. As expected, the polar solvation component partially counteracted these stabilizing interactions but did not reverse the overall affinity.

### 2.7. Molecular Dynamics Simulations

MD simulations were carried out to evaluate the dynamic stability of the protein–ligand complexes and their time-dependent conformational behavior. After system preparation, each complex was solvated in an orthorhombic TIP3P water box and neutralized to 0.15 M NaCl, and the initial system configuration are summarized in Table S3. Systems were equilibrated for 5 ns in the NVT ensemble and then subjected to a 100 ns production run in the NPT ensemble at 300 K and 1 bar. Analyses included root-mean-square deviation (RMSD), root-mean-square fluctuation (RMSF), and protein–ligand contact occupancies.

**Figure 8:**
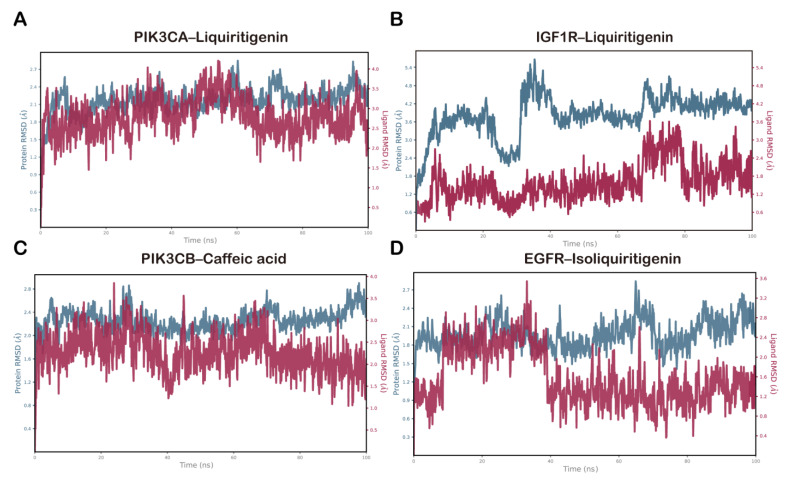
RMSD plots during molecular dynamics simulation for (**A**) PIK3CA–liquiritigenin, (**B**) IGF1R–liquiritigenin, (**C**) PIK3CB–caffeic acid, and (**D**) EGFR–isoliquiritigenin complexes.

Monitoring the RMSD of the protein provided insights into its structural stability throughout the simulation, while the ligand RMSD reflected whether the ligand remained within the binding pocket. For the PIK3CA–liquiritigenin complex, the protein backbone RMSD stabilized within 2.1–2.7 Å, and the ligand RMSD stabilized within 2.0–4.0 Å following the initial equilibration (Figure 8A). By contrast, the IGF1R–liquiritigenin complex showed a marked increase in protein RMSD during the first ~40 ns followed by a clear plateau, suggesting early conformational relaxation and subsequent stabilization. The ligand RMSD remained comparatively confined within the binding pocket throughout (Figure 8B). The PIK3CB–caffeic acid complex showed a backbone RMSD that stabilized between 2.0 and 2.8 Å during the later stage of the simulation, whereas the ligand equilibrated rapidly and was maintained within 1.2–2.5 Å, consistent with a tightly bound conformation (Figure 8C). For the EGFR–isoliquiritigenin complex, the protein RMSD varied smoothly between 1.8 and 2.7 Å, while the ligand RMSD stayed within 1.0–1.8 Å, consistent with a similarly stable binding mode (Figure 8D). Collectively, these RMSD profiles indicated that all four complexes reached equilibrium and maintained conformational stability over the 100 ns trajectories, supporting the credibility of the docking-derived binding poses.

The RMSF analysis was conducted to evaluate residue-level flexibility of the protein backbones over the 100 ns MD simulations. In all four complexes, the overall RMSF profiles indicated that most residues remained relatively stable, while pronounced fluctuations were primarily observed in loop regions and at the N- and C-termini.

**Figure 9:**
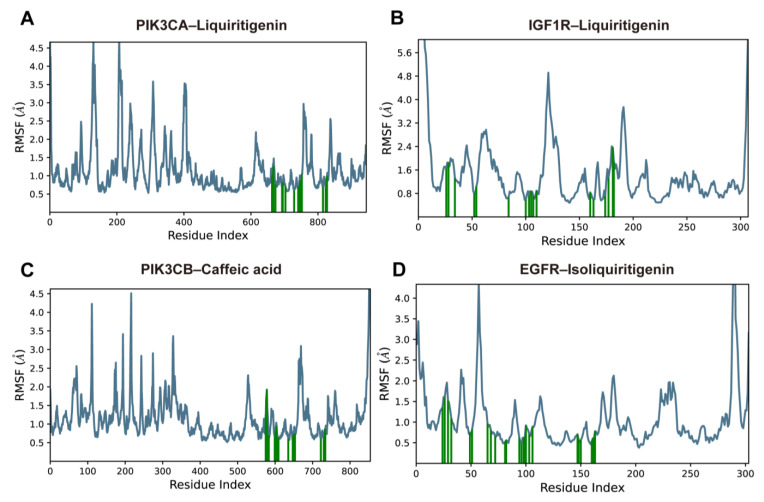
RMSF plots of protein Cα atoms over 100-ns MD simulations for (**A**) PIK3CA–liquiritigenin, (**B**) IGF1R–liquiritigenin, (**C**) PIK3CB–caffeic acid, and (**D**) EGFR–isoliquiritigenin complexes. The blue lines indicate the protein Cα RMSF; green vertical bars indicate residues directly involved in ligand contacts.

As shown in Figure 9, the peaks in the RMSF plots indicated the protein regions that fluctuated most during the simulations, and the residues involved in ligand contacts were marked with green vertical bars. Although several loop and terminal regions exhibited pronounced peaks approaching 4.0–5.0 Å, most ligand-contacting residues showed consistently moderate fluctuations, generally within 0.6–2.5 Å, which were substantially lower than those of the highly mobile loops. This pattern suggests that the binding sites maintained a balance between structural rigidity and local flexibility.

**Figure 10:**
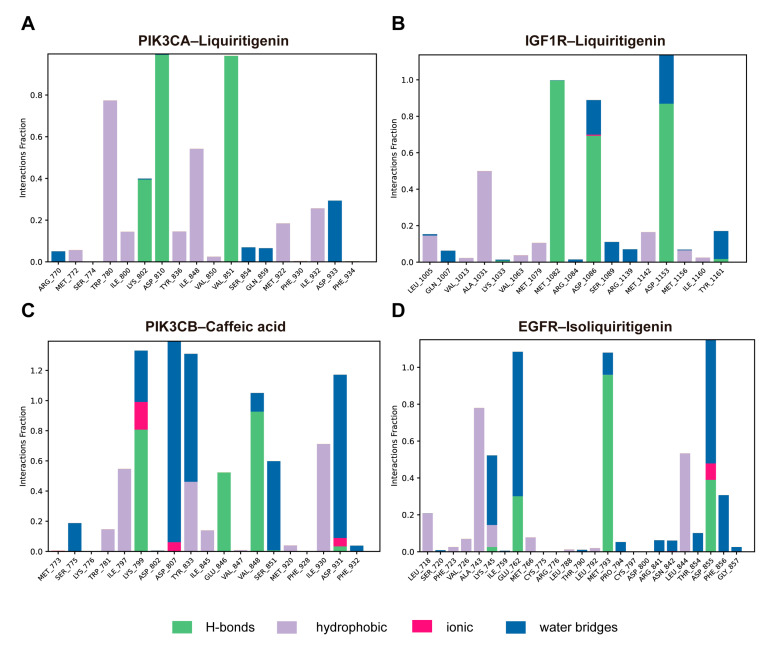
Protein–ligand interaction profiles over the simulation trajectory for (**A**) PIK3CA–liquiritigenin, (**B**) IGF1R–liquiritigenin, (**C**) PIK3CB–caffeic acid, and (**D**) EGFR–isoliquiritigenin complexes.

Protein–ligand interactions were monitored throughout the 100 ns simulations and classified into hydrogen bonds, hydrophobic interactions, ionic interactions, and water bridges. The interaction fraction denotes the proportion of simulation time during which a specific contact was maintained between a protein residue and the ligand. In the PIK3CA–liquiritigenin complex, key interacting residues included Trp780, Lys802, Asp810, Ile848, and Val851. These residues formed sustained contacts predominantly through hydrogen bonding and hydrophobic interactions (Figure 10A). Notably, Asp810 and Val851 displayed a hydrogen bond occupancy close to 1.0. Similarly, in the IGF1R–liquiritigenin complex, residues Ala1031, Met1082, Asp1086, and Asp1153 contributed prominently to ligand binding (Figure 10B). Several of these contacts persisted for most of the simulation, with interaction fractions approaching or exceeding 1.0, primarily through hydrogen bonds and water bridges. In the PIK3CB–caffeic acid complex, Lys799, Asp807, Tyr833, Val848, and Asp931 showed strong and persistent interactions, including hydrogen bonding, water bridging, and ionic contacts, with interaction fractions exceeding 1.0 (Figure 10C). For the EGFR–isoliquiritigenin complex, Ala743, and Leu844 were mainly involved in hydrophobic contacts. Water bridges and hydrogen bond also contributed modestly to pocket stabilization (Figure 10D). Overall, these results suggested that specific residues in each complex formed stable and, in some cases, multi-modal interactions with the ligand, playing crucial roles in maintaining binding affinity and structural integrity.

**Figure 11:**
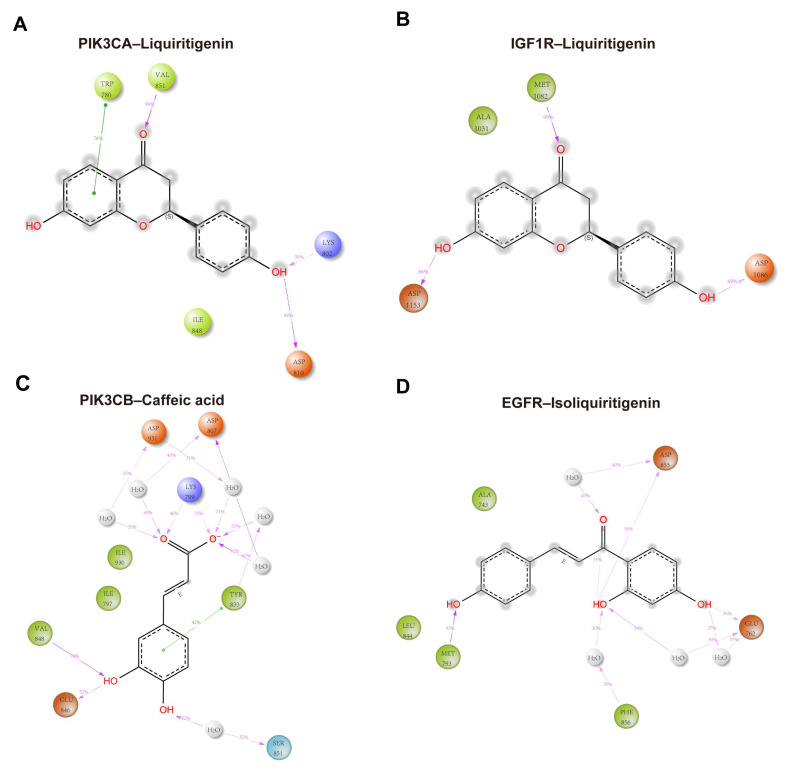
Schematic 2D interaction maps over the 100 ns MD simulations for (**A**) PIK3CA–liquiritigenin, (**B**) IGF1R–liquiritigenin, (**C**) PIK3CB–caffeic acid, and (**D**) EGFR–isoliquiritigenin complexes. Orange: negatively charged residues; purple: positively charged residues; light green: hydrophobic residues; light blue (cyan): polar residues; gray: water molecules; light-gray circles: solvent exposure. Solid green lines: π–π stacking interactions; purple dashed arrows: hydrogen bonds with their corresponding occupancy percentages.

Figure 11 illustrated schematic 2D interaction maps for the four protein–ligand complexes, highlighting interactions that persisted for >30% of the 100 ns molecular dynamics simulation. In the PIK3CA–liquiritigenin complex, the ligand formed direct hydrogen bonds with Lys802 (39%), Asp810 (99%), and Val851 (98%). In addition, Trp780 engaged in π–π stacking interactions with the aromatic system of liquiritigenin, reinforcing the binding through hydrophobic contacts (Figure 11A). These interactions collectively contributed to the stable anchoring of the ligand within the binding pocket. For the IGF1R–liquiritigenin complex, stable hydrogen bonding was observed with Met1082 (99%), Asp1086 (69%), and Asp1153 (86%) (Figure 11B). In the PIK3CB–caffeic acid complex, direct hydrogen bonds were formed with Lys799, Glu846, and Val848, while Asp807, Tyr833, Ser851, and Asp931 contributed mainly through water-bridged and π–π interactions, with interaction fractions ranging from 31% to 92% (Figure 11C). In the EGFR–isoliquiritigenin complex, the primary interaction involved hydrogen bond between Glu762, Met793, Asp 855, and Phe856 (Figure 11D). Together, these data revealed distinct binding profiles across the four complexes, with multiple key residues contributing to the stability and specificity of ligand recognition.

## 3. Discussion

This study systematically investigated the key bioactive constituents of ZXD and elucidated its antihypertensive mechanisms by integrating plasma UPLC–MS analysis, network pharmacology, molecular docking, and MD simulations. Initially, UPLC–MS was employed to identify the chemical constituents of ZXD, with particular emphasis on those absorbed into systemic circulation, as only bioavailable compounds are capable of exerting pharmacological effects in vivo [25]. Following oral administration of ZXD in rats, a total of 72 compounds were detected in plasma, comprising 14 prototype compounds and 58 metabolites. These metabolites, generated mainly through hydroxylation and sulfation, provide a realistic representation of the in vivo pharmacodynamics of ZXD.

Network pharmacology has emerged as a valuable tool for elucidating the active compounds, targets, and pharmacological mechanisms underlying complex TCM formulations [36]. In our analysis, bioavailable compounds from ZXD were associated with 149 hypertension-related targets, including pivotal nodes such as SRC, PTPN11, EGFR, PIK3CA, PIK3CB, HRAS, NRAS, PDGFRA, IGF1R, and MET, highlighting the multi-target characteristics of the prescription. Among them, PIK3CA and PIK3CB, key components of the PI3K/AKT signaling pathway, were particularly noteworthy. This pathway plays a central role in endothelial cell function, vascular smooth muscle cell (VSMC) proliferation, and inflammatory modulation, all of which are fundamental to blood pressure homeostasis [33,34]. These findings support the concept that complex herbal formulas exert their therapeutic effects through a multi-component, multi-target, and multi-pathway mechanism, as demonstrated in previous studies on TCM formulations [37,38].

KEGG pathway enrichment analysis further revealed that ZXD may exert antihypertensive effects via several key cascades, including AGE–RAGE [39], cAMP, and cGMP–PKG signaling pathways. Notably, several core targets of ZXD—such as PIK3CA, PIK3CB, and AKT1—were significantly enriched. The PI3K/AKT axis is pivotal in maintaining vascular endothelial integrity, regulating VSMC behavior, and preserving cardiac function [40]. Previous research has demonstrated that this pathway can mediate protection against Ang II–induced apoptosis through IGF1R–PI3K–AKT signaling, both in H9c2 cells and in spontaneously hypertensive rat models [41].

To validate the network-based predictions, molecular docking was conducted to assess the binding affinity between the key bioactive compounds and their corresponding protein targets. Docking results revealed favorable binding energies for key compounds such as liquiritigenin, caffeic acid, and isoliquiritigenin, with the most potent interactions reaching −8.725 kcal·mol^−1^. These results were further substantiated by 100 ns MD simulations, which confirmed the stability and persistence of the ligand–target interactions over time. These stable interactions suggest that the compounds could effectively modulate targets within the PI3K/AKT and EGFR-mediated signaling networks, reinforcing their potential contribution to the antihypertensive efficacy of ZXD.

Our previous studies demonstrated that ZXD reduced blood pressure in spontaneously hypertensive rats, and this antihypertensive effect is closely associated with modulation of the renin–angiotensin system [14]. Angiotensin II (Ang II), the primary bioactive peptide of the renin–angiotensin system, is a well-established contributor to hypertension through its roles in vascular contraction and adverse remodeling, which elevate peripheral resistance [42]. Src family kinases (SFK) play a critical role in Ang II-mediated hypertensive mechanisms. Targeted inhibition of SFK has been shown to significantly reduce systemic blood pressure in Ang II-treated mice [43]. In our study, SRC, an SFK family member, was identified as a key target of ZXD in hypertension treatment, showing strong interactions with the core component liquiritigenin. Moreover, clinical studies have shown that the combination of ZXD with amlodipine besylate significantly improves serum levels of renin, Ang II, and aldosterone in hypertensive patients [44], further corroborating the involvement of SRC-mediated pathways in the antihypertensive effects of ZXD.

**Figure 12:**
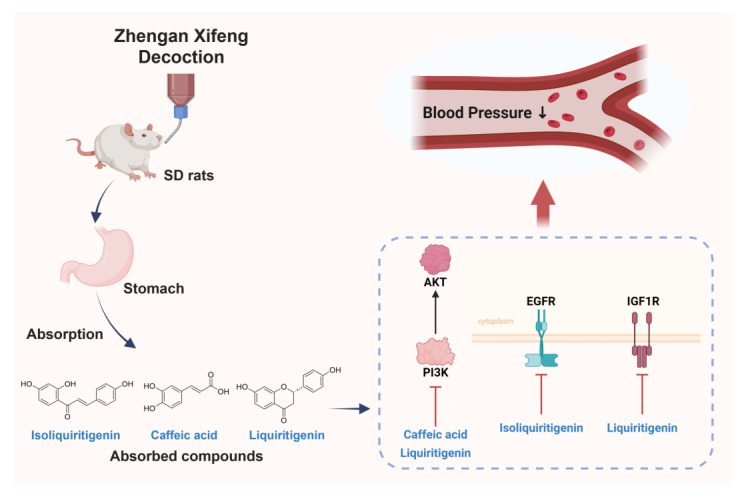
Integrative schematic of absorbed compounds and proposed mechanism underlying the antihypertensive effect of ZXD.

Collectively, this integrative analysis revealed that ZXD exerted its antihypertensive effects through a multi-component, multi-target, and multi-pathway strategy. By combining the identification of key bioavailable compounds with network pharmacology, molecular docking, and MD simulations, we provided comprehensive insights into the pharmacological mechanisms underlying ZXD’s therapeutic potential (Figure 12). Nevertheless, further experimental validation is necessary to confirm the predicted compound–target interactions and to elucidate the precise molecular mechanisms in vivo.

## 4. Materials and Methods

### 4.1. Chemicals and Reagents

ZXD is composed of twelve medicinal herbs: Niuxi (*Achyranthes bidentata* Blume), Daizheshi (Haematite), Shenglonggu (Fossilia OssiaMastodi), Muli (*Ostrea gigas* Thumb), Guiban (Carapax testudinis), Baishao (*Paeonia lactiflora* Pall), Xuanshen (*Scrophularia ningpoensis* Hemsl), Tiandong [*Asparagus cochinchinensis* (Lour.) Merr.], Chuanlianzi (*Melia toosendan*), Maiya (Fructus hordei germinatus), Yinchen (*Artemisia capillaris* Thunb), and Gancao (*Glycyrrhizae radix* et rhizoma) [9]. All herbs were provided and quality-checked by Tong Ren Tang Technologies Co., Ltd. (Beijing, China). LC–MS grade acetonitrile and formic acid were purchased from Fisher Scientific (Fair Lawn, NJ, USA), and distilled water was prepared using a Milli-Q system (MilliporeSigma, Burlington, MA, USA). All other reagents used in this study were of analytical grade and obtained from commercial sources.

### 4.2. Animals

Six specific pathogen-free (SPF) male Sprague Dawley (SD) rats (6–8 weeks old, 200 ± 20 g) were purchased from Beijing Vital River Laboratory Animal Technology Co., Ltd. (Beijing, China). Rats were housed under controlled conditions (25–28 °C) on a 12 h light/12 h dark cycle with ad libitum access to standard rodent chow and water. Rats underwent a 1-week acclimation period before any procedures. After a 1-week acclimation period, animals were randomly divided into two groups (*n* = 3 per group). This study was conducted according to the guidelines of the Declaration of Helsinki and was approved by the Animal Ethical Committee of Shanghai Wuchang Biotech Co., Ltd. (approval Number: WTPZ20240620001; approval date: 20 June 2024). All animal experiments were performed according to institutional guidelines for ethical animal studies.

### 4.3. Preparation of ZXD

The ZXD extract was prepared as previously described [15], and based on the composition and dosage proportions recorded in Yixue Zhongzhong Canxilu [7]. Briefly, four doses of medicinal materials, totaling 689 g, were prepared. Daizheshi, Shenglonggu, Muli, and Guiban were first decocted in water for 2 h. The remaining herbs were then added to eight times their volume of water, brought to a boil over high heat, and simmered for 30 min. The mixture was filtered, and the residue was further extracted two times with an additional eight volumes of water each time. The combined filtrates were concentrated to a smaller volume, then freeze-dried to yield 180.33 g of lyophilized powder.

### 4.4. Sample Preparation

#### 4.4.1. Preparation of ZXD Solution

To prepare the ZXD solution, 500 mg of lyophilized powder was dissolved in 10 mL of 50% methanol (*v*/*v*) by ultrasonication (40 kHz, 15 min) and centrifuged at 12,000 rpm for 5 min. A 5 µL aliquot of the supernatant was injected into the UPLC–Q–TOF/MS system.

#### 4.4.2. Plasma Sample Collection and Preparation

Blood samples were collected from the jugular vein of rats at predetermined time points—before administration and at 0, 0.25, 0.5, 1, 2, 4, and 6 h after administration of ZXD. At each time point, approximately 500 µL of whole blood was collected into tubes containing EDTA-K2. After centrifugation at 6000 rpm for 5 min at 4 °C, the plasma was collected. The plasma samples were pooled and subjected to protein precipitation using three volumes of methanol (*v*/*v*). Following vortexing, the samples were centrifuged at 12,000 rpm for 15 min at 4 °C, and the supernatant was evaporated under nitrogen. The residue was reconstituted in 100 µL of 50% methanol (*v*/*v*), vortexed for 3 min, the centrifuged again at 12,000 rpm for 15 min, and a 5 µL aliquot was injected into the UPLC–Q–TOF/MS system for analysis.

### 4.5. Chromatography and Mass Spectrometry Conditions

Chromatographic analysis was conducted on a Waters ACQUITY UPLC HSS T3 column (2.1 × 100 mm, 1.8 µm; Waters Corporation, Milford, MA, USA) maintained at 30 °C. The flow rate was set at 0.3 mL/min, with an injection volume of 5 µL. Detection was performed across a wavelength range of 190–400 nm using a Waters ACQUITY UPLC system (Waters Corporation, Milford, MA, USA). The mobile phase consisted of solvent A (0.1% formic acid in water, *v/v*) and solvent B (acetonitrile), with the following gradient elution program: 0–6 min, 0–8% B; 6–12 min, 8–18% B; 12–28 min, 18–35% B; 28–33 min, 35–45% B; 33–35 min, 45–95% B; 35–38 min, 95% B; 38–38.1 min, 95–0% B; 38.1–40 min, 0% B.

Mass spectrometry was performed on an AB Sciex Triple TOF^®^ 4600 mass spectrometer (SCIEX, Framingham, MA, USA) with an electrospray ionization (ESI) source, in both positive and negative modes. The TOF mass range was 50–1700 *m*/*z*, and the MS/MS range was 50–1250 *m*/*z*. Ion source gas 1 and gas 2 were set at 50 psi, curtain gas at 35 psi, and ion spray voltage at −4500 V (negative mode) and 5000 V (positive mode), with a declustering potential of 100 V. The ion source temperature was set to 500 °C.

### 4.6. Absorbed Compound Targets of ZXD

Identifying absorbed compounds provides insight into the potential bioactive components of ZXD that may contribute to its pharmacological effects. Simplified Molecular Input Line Entry System (SMILES) representations of the absorbed compounds were retrieved from the PubChem database (https://pubchem.ncbi.nlm.nih.gov/, accessed on 5 September 2024) [26]. These absorbed compounds were then used for target prediction using the TCMSP (https://tcmsp-e.com/tcmsp.php, accessed on 8 September 2024) [28] and SwissTargetPrediction (http://swisstargetprediction.ch, accessed on 8 September 2024) [27] databases. In the Swiss Target Prediction database, targets were screened using a probability threshold of >0.

### 4.7. Collection of Potential Targets for ZXD

To delineate potential therapeutic targets of ZXD for hypertension, the keyword “Hypertension” and the filter “Homo sapiens” were applied in searches of the GeneCards database (https://www.genecards.org, accessed on 3 October 2024) [29] and the Online Mendelian Inheritance in Man (OMIM) database (https://omim.org, accessed on 3 October 2024) [30]. These searches produced a comprehensive list of hypertension-associated targets.

The targets from both databases were consolidated, and duplicates removed, to form a refined set of hypertension-associated targets. This refined list was compared with the predicted targets of ZXD’s absorbed compounds. The Draw Venn Diagram tool (https://bioinformatics.psb.ugent.be/webtools/Venn/, accessed on 29 October 2024) [45] was employed to identify intersecting targets, representing potential therapeutic targets of ZXD for hypertension. This intersection of disease and compound-predicted targets enabled focused network pharmacology analysis.

### 4.8. Construction of the Absorbed Compound–Target Network

To visualize interactions between ZXD’s absorbed compounds and hypertension-related targets, a compound–target interaction network was constructed using Cytoscape 3.10.2 [31]. Nodes represented either compounds or target proteins, while edges indicated compound–target interactions. Topological properties, including closeness centrality, betweenness centrality, and degree, were calculated using the “Network Analyzer” tool in Cytoscape. This network analysis identified primary active compounds in ZXD with potential antihypertensive activity.

### 4.9. Construction and Analysis of the Protein–Protein Interaction Network

The intersecting targets identified for ZXD in the treatment of hypertension were imported into the STRING 11.5 database (https://www.string-db.org/, accessed on 29 October 2024) [32] to analyze protein–protein interactions (PPI) among these targets. The species was set to “*Homo sapiens*” with a confidence level threshold of >0.9. The resulting PPI network data were imported into Cytoscape 3.10.2 software, and the cytoHubba plugin was used to identify hub targets using the Maximal Clique Centrality (MCC) metric. In this study, we operationally defined “core targets” as hub nodes ranked in the top 10 by MCC under these settings, a convention frequently adopted in network-pharmacology analyses [46,47,48]. These top 10 nodes were selected and plotted to highlight key molecular targets within the network, with node color encoding the MCC score (red → yellow for higher → lower).

### 4.10. GO and KEGG Enrichment Analyses

To investigate the mechanisms by which ZXD may exert therapeutic effects on hypertension, GO functional annotation and KEGG pathway enrichment analyses were conducted using the Metascape database (http://metascape.org/gp/index.html, accessed on 6 November 2024) [49]. Analyses were performed specifically for human species, focusing on BP, CC, MF, and KEGG signaling pathways relevant to ZXD and hypertension.

Enrichment results were filtered using a false discovery rate threshold of less than 0.01 and a minimum count of three to ensure significance. The top ten GO terms and top 20 KEGG pathways, based on count values, were visualized using the Bioinformatics platform (https://www.bioinformatics.com.cn, accessed on 6 November 2024) to illustrate the primary biological pathways and molecular functions associated with the therapeutic targets of ZXD.

### 4.11. Molecular Docking Analysis

Molecular docking studies were conducted to predict the binding interactions between the core absorbed components of ZXD and the ten core target proteins. The crystal structures of target proteins were retrieved from the RCSB Protein Data Bank (https://www.rcsb.org/, accessed on 21 November 2024). Protein preparation was carried out using the Protein Preparation Wizard module in Maestro (Schrödinger, 2021), which included preprocessing steps such as removal of water molecules, addition of hydrogens, assignment of protonation states, and energy minimization. Subsequently, the receptor grid was generated based on the co-crystallized ligand position. The 2D structure files (SDF format) of the active compounds were downloaded from the PubChem database, and preprocessed using the LigPrep module in Schrödinger to generate low-energy 3D conformers with appropriate protonation states and stereochemistry.

Docking was performed using Glide in standard precision (SP) mode, and for each protein–ligand pair, the top four docking poses with the highest GlideScores were retained for further analysis. The protein–ligand interaction profiles were visualized and analyzed using PyMOL and the Ligand Interaction Diagram tool in Maestro to identify key residues involved in binding.

### 4.12. MM-GBSA Binding Free Energy Estimation

Following molecular docking, the four selected protein–ligand complexes were subjected to Prime MM-GBSA to estimate binding free energies (Δ*G*_bind_). Calculations employed the OPLS4 force field together with the VSGB implicit solvation model. For each system, the free energies of the complex, the isolated receptor, and the isolated ligand were evaluated (standard single-trajectory approximation), and Δ*G*_bind_ was obtained as:

$${\Delta G}_{bind}=G_{complex}-(G_{protein}+G_{ligand})$$

The total free energy was decomposed as:

$$G=E_{MM}+G_{solv}-T\Delta S$$

where $E_{MM}$ comprises molecular-mechanics terms (e.g., van der Waals and electrostatic/Coulombic interactions), and $G_{solv}$ includes the polar (GB/VSGB) and non-polar (SASA/lipophilic) contributions. Unless otherwise specified, the entropic term TΔS was not explicitly calculated. Therefore, MM-GBSA values are interpreted comparatively (for ranking) rather than as absolute affinities. All energies are reported in kcal·mol^−1^.

### 4.13. Molecular Dynamics Simulation

To further assess the structural stability and dynamic behavior of the four protein–ligand complexes, MD simulations were carried out in Maestro (Schrödinger, 2021). Each docked complex was prepared with the System Builder module and solvated in an orthorhombic periodic water box using the TIP3P model. Counterions (Na^+^/Cl^−^) were added to neutralize the total charge and to achieve a bulk salt concentration of 0.15 M. The systems were then energy-minimized under the OPLS4 force field to relieve steric clashes (see Table S3 for system composition).

Equilibration was performed for 5 ns in the NVT ensemble to stabilize the temperature, followed by a 100 ns production run in the NPT ensemble at 300 K and 1 bar. Trajectories were analyzed with the Simulation Interaction Diagram panel to compute root-mean-square deviation (RMSD), root-mean-square fluctuation (RMSF), and key protein–ligand interactions for evaluating binding stability.

## 5. Conclusions

In summary, this study systematically elucidated the key bioactive constituents and underlying therapeutic mechanisms of ZXD against hypertension through an integrated strategy combining plasma UPLC–MS analysis, network pharmacology, molecular docking, MM-GBSA binding free energy estimation, and MD simulations. A total of 72 absorbed components were identified, including 14 prototype compounds and 58 metabolites. Network pharmacology revealed ten key bioactive compounds (e.g., liquiritigenin, isoliquiritigenin, and caffeic acid) and ten core targets (such as PIK3CA, PIK3CB, EGFR, and IGF1R). Functional enrichment implicated cardiovascular, metabolic, and stress-response pathways in the antihypertensive effects of ZXD. Molecular docking demonstrated strong binding affinities between representative compounds and core targets, with binding energies as low as −8.725 kcal·mol^−1^. Prime MM-GBSA supported the stability of the predicted complexes. These results were further validated by MD simulations, which confirmed the structural stability and sustained interactions of the protein–ligand complexes throughout the 100 ns simulation period. Collectively, these findings provide comprehensive mechanistic insights into the multi-component, multi-target, and multi-pathway pharmacological effects of ZXD, highlighting its therapeutic potential in the management of hypertension.

## Figures and Tables

**Table 1:** Characterization of 13 newly identified chemical constituents in ZXD by UPLC–MS.

No.	t_R_ (min)	Element Composition	Ion Mode	Theoretical *m*/*z*	Observed *m*/*z*	Error (ppm)	Fragment Ions	Compounds	Sources
1	5.80	C_21_H_32_O_14_	ESI-	553.1774	553.1793	3.4	507.1735, 147.0450, 89.0236	6-*O*-β-glucopyranosylaucubin	Xuanshen
2	8.58	C_16_H_18_O_9_	ESI-	353.0873	353.0882	2.5	353.0878, 191.0556, 179.0345, 135.0446, 134.0369, 85.0291	chlorogenic acid	Yinchen
3	8.71	C_16_H_24_O_8_	ESI-	389.1453	389.1451	−0.5	389.1451, 343.1408, 181.0869, 161.0462, 151.0768, 136.0533, 109.0666	mudanpioside F	Baishao
4	10.02	C_23_H_28_O_13_S	ESI-	543.1178	543.1177	−0.2	543.1193, 421.0798, 259.0273, 121.0292	paeoniflorin sulfite	Baishao
5	10.49	C_16_H_18_O_9_	ESI-	353.0873	353.0886	3.7	192.0599, 191.0568	neochlorogenic acid	Yinchen
6	10.90	C_16_H_18_O_9_	ESI-	353.0873	353.0886	3.7	191.0545, 179.0341, 173.0441, 135.0443, 93.0339	cryptochlorogenic acid	Yinchen
7	17.42	C_23_H_28_O_11_	ESI-	525.1608	525.1633	4.8	479.1573, 283.0818, 121.0296, 77.0392	mudanpioside I	Baishao
8	18.15	C_36_H_48_O_19_	ESI-	783.2717	783.2721	0.5	783.2736, 607.2253, 175.0400	angoroside C	Xuanshen
9	19.19	C_22_H_22_O_9_	ESI-	475.1246	475.1261	3.2	267.0655;252.0411	formononetin glucoside	Gancao
10	20.60	C_21_H_34_O_10_	ESI-	491.2134	491.2142	1.6	491.2103, 445.2060, 293.0860, 191.0556, 89.0235, 59.0127	pinen-10-yl vicianoside	Baishao
11	29.78	C_42_H_62_O_17_	ESI-	837.3914	837.3935	2.5	837.3963, 351.0556	licorice saponin G2	Gancao
12	30.65	C_42_H_62_O_17_	ESI-	837.3927	837.3929	0.2	837.3953, 351.0576	uralsaponin U	Gancao
13	36.23	C_20_H_16_O_6_	ESI-	351.0869	351.0878	2.6	352.0877, 283.0960, 199.0754	licoisoflavone B	Gancao

**Table 2:** Network pharmacology analysis results.

No.	Compounds	Sources	Average Shortest Pathlength	Betweenness Centrality	Degree
D11	isoliquiritigenin	Gancao	2.378698225	0.193318	41
D3	liquiritigenin	Gancao	2.378698225	0.192043	41
D8	formononetin	Gancao	2.414201183	0.184771	39
D6	caffeic acid	Yinchen	2.485207101	0.127596	32
P7	β-ecdysterone	Niuxi	2.674556213	0.146296	28
D1	paeonol	Baishao	2.603550296	0.103937	25
P5	albiflorin	Baishao	2.591715976	0.101707	25
P14	glycyrrhizic acid	Gancao	2.686390533	0.040437	19
P12	licorice saponin G2	Gancao	2.816568047	0.029340	19
P13	uralsaponin U	Gancao	2.816568047	0.029340	19

**Table 3:** Docking results of the key bioactive compounds with core targets.

No.	Key Compounds	Core Targets	PDB ID	Binding Energy (kcal·mol^−1^)
D3	liquiritigenin	PIK3CA	8EXL	−8.725
		IGF1R	5FXS	−8.689
		PIK3CB	4BFR	−8.133
		SRC	4U5J	−7.763
		MET	3DKC	−7.501
P5	albiflorin	EGFR	7U99	−6.017
D6	caffeic acid	PIK3CB	4BFR	−7.113
D11	isoliquiritigenin	EGFR	7U99	−7.055
		IGF1R	5FXS	−7.777
D8	formononetin	EGFR	7U99	−7.084

## Data Availability

Data are contained within the article and Supplementary Materials.

## Supplementary Materials

The following supporting information can be downloaded at: https://www.mdpi.com/article/10.3390/ph18101493/s1, Figure S1: Chemical profiling of absorbed components from ZXD by UPLC–MS; Table S1: Characterization of prototype compounds identified from ZXD-medicated plasma by UPLC–MS; Table S2: Characterization of metabolites identified from ZXD-medicated plasma by UPLC–MS. Table S3: Initial configurations of molecular dynamics simulation systems.
