# Supplementary material for: Bioactive Constituents and Antihypertensive Mechanisms of Zhengan Xifeng Decoction: Insights from Plasma UPLC–MS, Network Pharmacology and Molecular Dynamics Simulations
Source: Pharmaceuticals (Basel). 2025 Oct 4;18(10):1493. doi: 10.3390/ph18101493 (PMC12567505; doi:10.3390/ph18101493)
Supplement: Supplementary file 1 [file pharmaceuticals-18-01493-s001.zip › pharmaceuticals-3857907-supplementary.pdf]

Bioactive constituents and antihypertensive mechanisms of Zhengan Xifeng Decoction: insights from plasma UPLC–MS, network pharmacology, and molecular dynamics simulations

Yu Wang [1,2,3,†], Yiyi Li [4,†], Zhuoying Lin [4], Niping Li [4], Qiuju Zhang [1,2,3], Shuangfang Liu [2,3,5], Meilong Si [2,3,5] and Hua Jin [2,3,5,*]

1 School of Basic Medicine, Gansu University of Chinese Medicine, Lanzhou 730000, China

2 Key Laboratory of Dunhuang Medicine, Ministry of Education, Lanzhou 730000, China

3 Key Laboratory of Traditional Chinese Herbs and Prescription Innovation and Transformation of Gansu Province, Lanzhou 730000, China

4 College of Pharmacy, Jinan University, Guangzhou 510632, China

5 Clinical of Traditional Chinese Medicine, Gansu University of Chinese Medicine, Lanzhou 730000, China

* Correspondence: E-mail: lanzhoujinhua@126.com (H.J.)

† These authors contributed equally to this work

Figure captions

Figure S1. Chemical profiling of absorbed components from ZXD by UPLC–MS.

Table lists

Table S1. Characterization of prototype compounds identified from ZXD-medicated plasma by UPLC–MS.

Table S2. Characterization of metabolites identified from ZXD-medicated plasma by UPLC–MS.

Table S3. Initial configurations of molecular dynamics simulation systems

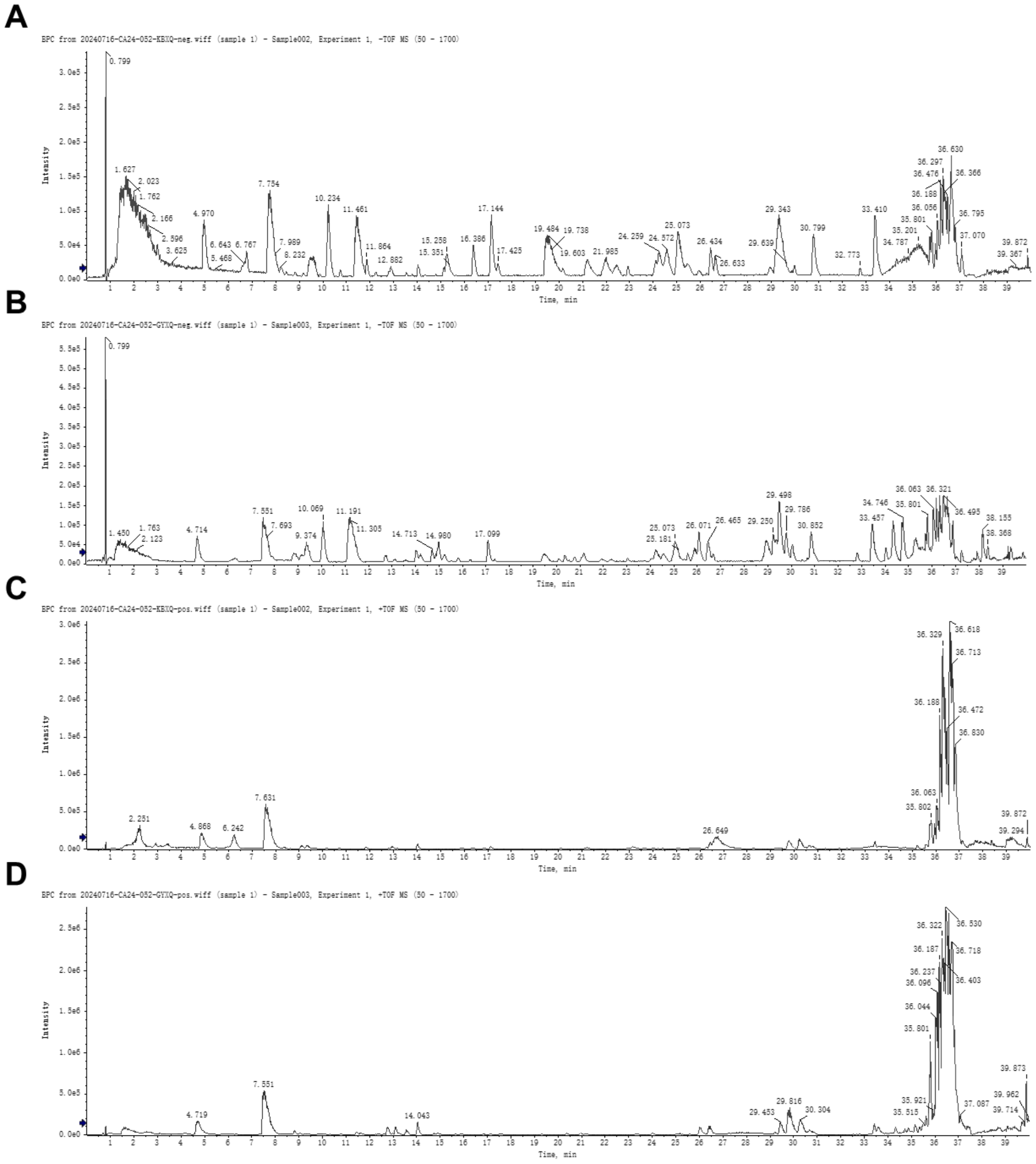

Figure S1. Chemical profiling of absorbed components from ZXD by UPLC–MS. (A) Base peak ion (BPI) chromatogram of control plasma in negative ion mode. (B) BPI chromatogram of ZXD-medicated plasma in negative ion mode. (C) BPI chromatogram of control plasma in positive ion mode. (D) BPI chromatogram of ZXD-medicated plasma in positive ion mode.

Table S1. Characterization of prototype compounds identified from ZXD-medicated plasma by UPLC–MS.

No.	t_R (min)	Compounds	Ion mode	Observed *m/z*	Theoretical *m/z*	Element composition	Error (ppm)	Fragment ions	Sources [a]
P1	5.65	6-*O*-β-glucopyranosylaucubin	ESI-	553.1781	553.1774	$C_{21}H_{32}O_{14}$	1.3	553.2481, 507.1760, 455.0024, 147.0452, 89.0257	Xuanshen
P2	7.12	harpagide	ESI-	409.1344	409.1352	$C_{15}H_{24}O_{10}$	-2.0	363.1286, 201.0759, 183.0662, 139.0399	Xuanshen
P3	8.63	mudanpioside F	ESI-	389.1444	389.1453	$C_{16}H_{24}O_{8}$	-2.3	389.1414, 343.1426, 181.0873, 161.0439, 151.0763, 136.0540, 109.0654, 96.9595	Baishao
P4	10.25	paeoniflorin sulfite	ESI-	543.1171	543.1178	$C_{23}H_{28}O_{13}S$	-1.3	543.1171, 421.0811, 341.9990, 259.0242, 121.0301	Baishao
P5	12.28	albiflorin	ESI-	525.1614	525.1608	$C_{23}H_{28}O_{11}$	1.1	525.1618, 479.1560, 357.1226, 283.0817, 121.0290, 77.0388	Baishao
P6	12.95	paeoniflorin	ESI-	525.1605	525.1608	$C_{23}H_{28}O_{11}$	-0.6	525.1590, 479.1534, 449.1440, 327.1067, 165.0553, 121.0290, 77.0391	Baishao
P7	14.88	β-ecdysterone	ESI-	525.3085	525.3069	$C_{27}H_{44}O_{7}$	3.0	525.3064, 479.2996, 301.1966, 269.0436, 159.1019	Niuxi
P8	17.48	mudanpioside I	ESI-	525.1615	525.1608	$C_{23}H_{28}O_{11}$	1.3	525.1646, 357.1197, 283.0847, 121.0297, 77.0405	BaiShao
P9	17.93	angoroside C	ESI-	783.2724	783.2717	$C_{36}H_{48}O_{19}$	0.9	783.2703, 607.2413, 175.0435, 149.0425, 59.0147	Xuanshen
P10	19.04	formononetin glucoside	ESI-	475.1254	475.1246	$C_{22}H_{22}O_{9}$	1.7	475.1185, 299.0578, 267.0661, 113.0235, 57.0441	Gancao
P11	20.43	pinen-10-yl vicianoside	ESI-	491.2127	491.2134	$C_{21}H_{34}O_{10}$	-1.4	491.2233, 453.0815, 445.2065, 293.0896, 237.0574, 191.0565, 135.0106, 89.0259, 59.0131	Baishao
P12	29.72	licorice saponin G2	ESI-	837.3923	837.3914	$C_{42}H_{62}O_{17}$	1.1	837.3898, 351.0549	Gancao
P13	30.56	uralsaponin U	ESI-	837.3927	837.3914	$C_{42}H_{62}O_{17}$	1.6	837.3928, 351.0556	Gancao
P14	31.60	glycyrrhizic acid	ESI-	821.3991	821.3965	$C_{42}H_{62}O_{16}$	3.2	821.3967, 351.0566, 193.0354	Gancao

Table S2. Characterization of metabolites identified from ZXD-medicated plasma by UPLC–MS.

No.	prototype compounds	metabolites	t_R (min)	Ion mode	Observed *m/z*	Theoretical *m/z*	Element composition	Error (ppm)	Fragment ions	Metabolic pathways
D1	paeonol	M1	15.02	ESI-	261.0076	261.0074	$C_9H_{10}O_7S$	0.8	260.0596, 181.0503, 166.0271, 151.0050, 107.0138	hydroxylation+ sulfation
P14	glycyrrhizic acid	M2	37.30	ESI-	469.3332	469.3323	$C_{30}H_{46}O_4$	1.9	469.3300, 425.3411	hydrolysis
D2	liquiritin	M3	11.31	ESI-	593.1502	593.1512	$C_{27}H_{30}O_{15}$	-1.7	593.1505, 573.1371, 417.1176, 255.0653	glucuronidation
		M4	14.25	ESI-	593.1518	593.1512	$C_{27}H_{30}O_{15}$	1.0	593.1531, 417.1156, 255.0689, 187.0075	glucuronidation
D3	liquiritigenin	M5	11.66	ESI-	513.0696	513.0708	$C_{21}H_{22}O_{13}S$	-2.3	513.0652, 433.1157, 337.0401, 216.9802, 137.0271, 113.0309	hydrogenation+ glucuronidation+ sulfation
		M6	15.00	ESI-	529.0668	529.0652	$C_{21}H_{22}O_{14}S$	3.0	431.0944, 255.0695, 96.9602	hydrogenation+ hydroxylation+ glucuronidation+ sulfation
		M7	23.04	ESI-	445.1139	445.1140	$C_{22}H_{22}O_{10}$	-0.2	445.1120, 269.0796, 254.0579, 175.0227, 113.0245	methylation+ glucuronidation
		M8	19.87	ESI-	335.0226	335.0231	$C_{15}H_{12}O_7S$	-1.5	335.0268, 255.0660, 135.0079, 119.0500, 91.0187	sulfation
		M9	27.65	ESI-	335.0233	335.0231	$C_{15}H_{12}O_7S$	0.6	335.2222, 255.0662, 135.0083, 119.0494, 91.0174	sulfation
		M10	14.72	ESI-	431.0984	431.0984	$C_{21}H_{20}O_{10}$	0.0	431.0978, 255.0658, 175.0244, 135.0093, 113.0244	glucuronidation
		M11	18.60	ESI-	511.0556	511.0552	$C_{21}H_{20}O_{13}S$	0.8	511.0577, 431.0959, 255.0658, 135.0108	glucuronidation+ sulfation
		M12	17.36	ESI-	447.0925	447.0933	$C_{21}H_{20}O_{11}$	-1.8	447.0916, 271.0611, 175.0258, 151.0029, 113.0247	hydroxylation+ glucuronidation
		M13	17.53	ESI-	447.0936	447.0933	$C_{21}H_{20}O_{11}$	0.7	447.0953, 271.0610, 175.0258, 151.0035, 113.0244	hydroxylation+

									glucuronidation
		M14	16.40	ESI-	527.0491	527.0501	$C_{21}H_{20}O_{14}S$	-1.9	527.0544, 447.0918, 351.0483, 271.0602, 151.0062	hydroxylation+ glucuronidation+ sulfation
D4	licoisoflavone B	M15	8.62	ESI-	379.1174	379.1187	$C_{22}H_{20}O_6$	-3.4	379.1151, 151.0772	dimethylation
		M16	26.64	ESI-	527.1203	527.1195	$C_{26}H_{24}O_{12}$	1.5	527.123, 351.0878, 175.0233, 113.0236	glucuronidation
		M17	28.74	ESI-	527.1204	527.1195	$C_{26}H_{24}O_{12}$	1.7	527.1212, 351.0864, 283.0946, 199.0732, 151.0032	glucuronidation
		M18	31.14	ESI-	527.1196	527.1195	$C_{26}H_{24}O_{12}$	0.2	527.2149, 481.2053, 351.0863, 283.0960, 265.0879	glucuronidation
		M19	23.03	ESI-	513.1028	513.1039	$C_{25}H_{22}O_{12}$	-2.1	513.1036, 445.1133, 269.0772, 254.0571, 175.0243	glucuronidation+ demethylation
D5	harpagenin	M20	13.79	ESI-	165.0562	165.0557	$C_9H_{10}O_3$	3.0	147.0470, 121.0664, 119.0484, 106.0416, 103.0534	didehydration
D6	caffeic acid	M21	11.46	ESI-	289.0386	289.0387	$C_{11}H_{14}O_7S$	-0.3	289.0416, 209.0798, 191.0698, 147.0799, 101.0239	hydrogenation+ dimethylation+ sulfation
		M22	14.40	ESI-	261.0066	261.0074	$C_9H_{10}O_7S$	-3.1	260.0596, 181.0503, 166.0271, 151.0050, 107.0138	hydrogenation+ sulfation
		M23	12.14	ESI-	357.0820	357.0822	$C_{15}H_{18}O_{10}$	-0.6	193.0537, 181.0508, 166.0283, 151.0042, 113.0232, 85.0288	hydrogenation+ glucuronidation
		M24	10.88	ESI-	369.0821	369.0827	$C_{16}H_{18}O_{10}$	-1.6	193.0493, 178.0276, 134.0378, 113.0233	methylation+ glucuronidation
		M25	14.91	ESI-	369.0839	369.0827	$C_{16}H_{18}O_{10}$	3.3	369.0787, 193.0508, 161.0231, 133.0299, 113.0246	methylation+ glucuronidation
		M26	8.61	ESI-	355.0660	355.0671	$C_{15}H_{16}O_{10}$	-3.1	355.0880, 179.0354, 135.0445	glucuronidation
		M27	10.25	ESI-	355.0656	355.0671	$C_{15}H_{16}O_{10}$	-4.2	355.0692, 311.0838, 179.0350, 135.0462	glucuronidation
		M28	11.58	ESI-	215.0024	215.0020	$C_8H_8O_5S$	1.9	135.0450, 120.0217, 92.0279, 81.9526	(-CO_2)+sulfation

D7	chlorogenic acid	M29	9.49	ESI-	355.1032	355.1035	$C_{16}H_{20}O_9$	-0.8	355.0950, 228.9498, 179.0707, 164.0467, 146.0377, 135.0450	hydrogenation
		M30	14.57	ESI-	179.0355	179.0350	$C_9H_8O_4$	2.8	143.8634;99.9278;77.0404	hydrolysis
		M31	14.27	ESI-	193.0505	193.0506	$C_{10}H_{10}O_4$	-0.5	178.0250, 134.0364, 61.9879	hydrolysis+ methylation
		M32	10.69	ESI-	163.0405	163.0401	$C_9H_8O_3$	2.5	162.8382, 119.0500, 93.0344	hydrolysis+ dehydroxylation
D8	formononetin	M33	19.05	ESI-	475.1254	475.1246	$C_{23}H_{24}O_{11}$	1.7	475.1185, 299.0578, 267.0661	dihydroxylation+ glucuronidation
		M34	28.10	ESI-	347.0234	347.0231	$C_{16}H_{12}O_7S$	0.9	347.0229, 267.0653, 252.0414, 223.0376	sulfation
		M35	19.29	ESI-	443.0979	443.0984	$C_{22}H_{20}O_{10}$	-1.1	443.0987, 267.0649, 252.0411, 175.0243, 113.0246	glucuronidation
		M36	16.74	ESI-	459.0925	459.0933	$C_{22}H_{20}O_{11}$	-1.7	459.0902, 283.0597, 268.0368, 113.0250	hydroxylation+ glucuronidation
D9	hydroxytyrosol	M37	10.32	ESI-	247.0280	247.0282	$C_9H_{12}O_6S$	-0.8	247.0277, 167.0709, 152.0481, 137.0607, 122.0365	methylation +sulfation
		M38	7.08	ESI-	233.0129	233.0125	$C_8H_{10}O_6S$	1.7	153.0564, 135.0457, 120.0223, 109.0290	sulfation
		M39	7.53	ESI-	233.0112	233.0125	$C_8H_{10}O_6S$	-5.6	233.0149, 153.0566, 145.1006, 123.0455	sulfation
		M40	8.29	ESI-	233.0128	233.0125	$C_8H_{10}O_6S$	1.3	233.0128, 153.0556, 135.0456, 123.0450	sulfation
		M41	6.08	ESI-	329.0878	329.0878	$C_{14}H_{18}O_9$	0.0	329.0866, 241.0602, 153.0529, 135.0442, 85.0291, 71.0141	glucuronidation
P6	paeoniflorin	M42	2.92	ESI-	421.1345	421.1351	$C_{16}H_{24}O_{10}$	-1.4	421.0893, 375.1305, 345.1200, 180.0646, 165.0562	debenzoyl
		M43	3.45	ESI-	421.1314	421.1351	$C_{16}H_{24}O_{10}$	-8.8	421.1323, 375.1310, 345.1187, 213.0764, 195.0675, 183.0658, 151.0769	debenzoyl
		M44	4.58	ESI-	421.1352	421.1351	$C_{16}H_{24}O_{10}$	0.2	375.1247, 345.1161, 213.0734, 195.0646, 183.0652, 151.0770	debenzoyl
		M45	8.27	ESI-	421.1350	421.1351	$C_{16}H_{24}O_{10}$	-0.2	183.0615, 151.0440, 135.0446, 113.0241	debenzoyl

		M46	9.22	ESI-	389.1463	389.1453	$C_{17}H_{26}O_{10}$	2.6	351.0317, 291.0845	($-C_7H_4O$)+ methylation
		M47	13.99	ESI-	357.1196	357.1191	$C_{16}H_{22}O_9$	1.4	357.1197, 113.0230, 85.0298, 75.0091	($-C_7H_4O$)+ dehydration
		M48	6.61	ESI-	435.1513	435.1508	$C_{17}H_{26}O_{10}$	1.1	435.1322, 389.1462, 227.0953, 177.0583, 123.0432	debenzoyl+ methylation
		M49	12.95	ESI-	449.1461	449.1453	$C_{22}H_{26}O_{10}$	1.8	449.1603, 165.0554, 121.0290	dehydroxylation+ demethylation
D10	neochlorogenic acid	M50	13.08	ESI-	163.0404	163.0401	$C_9H_8O_3$	1.8	162.0553, 119.0500, 93.0335	hydrolysis+ dehydroxylation
D11	isoliquiritigenin	M51	19.51	ESI-	433.1133	433.1140	$C_{21}H_{22}O_{10}$	-1.6	433.1144, 257.0805, 175.0234, 151.0392, 113.0228, 85.0288	hydrogenation+ glucuronidation
		M52	19.96	ESI-	433.1142	433.1140	$C_{21}H_{22}O_{10}$	0.5	433.1158, 257.0810, 175.0254, 151.0408, 113.0255	hydrogenation+ glucuronidation
		M53	17.70	ESI-	513.0713	513.0708	$C_{21}H_{22}O_{13}S$	1.0	513.0751, 433.1141, 337.0366, 257.0822, 175.0240, 151.0394	hydrogenation+ glucuronidation+ sulfation
		M54	18.95	ESI-	513.0710	513.0708	$C_{21}H_{22}O_{13}S$	0.4	513.0615, 433.1151, 337.0381, 257.0817, 175.0252, 151.0395	hydrogenation+ glucuronidation+ sulfation
		M55	20.38	ESI-	529.0673	529.0652	$C_{21}H_{22}O_{14}S$	4.0	431.0957, 255.0690, 96.9602	hydrogenation+ hydroxylation+ glucuronidation+ sulfation
		M56	19.79	ESI-	431.0984	431.0984	$C_{21}H_{20}O_{10}$	0.0	431.2099, 255.0659, 135.0076, 119.0495	glucuronidation
		M57	20.37	ESI-	431.0981	431.0984	$C_{21}H_{20}O_{10}$	-0.7	431.1007, 255.0667, 175.0251, 135.0088, 119.0507, 113.0246, 85.0292	glucuronidation
D12	cryptochlorogenic acid	M58	8.18	ESI-	163.0395	163.0401	$C_9H_8O_3$	-3.7	162.8429, 119.0519	hydrolysis+ dehydroxylation

Table S3. Initial configurations of molecular dynamics simulation systems

System (protein-ligand complex)	Total atoms	Total residues	Protein atoms	Protein residues	Ligand (atoms)	Solvent model	Water molecules (TIP3P)	Box type	Salt (NaCl)	Na^+ ions	Cl^- ions
PIK3CA-liquiritigenin	108,741	32,159	15,415	945	31	TIP3P	93,123	Orthorhombic	0.15 M	86	86
IGF1R-liquiritigenin	39,920	12,030	4,880	308	31	TIP3P	34,932	Orthorhombic	0.15 M	44	33
PIK3CB-caffeic acid	96,545	28,518	13,865	855	20	TIP3P	82,497	Orthorhombic	0.15 M	86	77
EGFR-isoliquiritigenin	36,301	10,807	4,890	304	31	TIP3P	31,317	Orthorhombic	0.15 M	34	29
